# Insights from Traditional Chinese Medicine for Restoring Skin Barrier Functions

**DOI:** 10.3390/ph17091176

**Published:** 2024-09-05

**Authors:** Jieyi Yang, Jiageng Guo, Peiling Tang, Shidu Yan, Xiaodong Wang, Huaying Li, Jinling Xie, Jiagang Deng, Xiaotao Hou, Zhengcai Du, Erwei Hao

**Affiliations:** 1 Guangxi Key Laboratory of Efficacy Study on Chinese Materia Medica, Guangxi University of Chinese Medicine, Nanning 530000, China; 2 Guangxi Collaborative Innovation Center of Study on Functional Ingredients of Agricultural Residues, Guangxi University of Chinese Medicine, Nanning 530000, China; 3 Guangxi Key Laboratory of TCM Formulas Theory and Transformation for Damp Diseases, Guangxi University of Chinese Medicine, Nanning 530000, China

**Keywords:** traditional Chinese medicine (TCM), skin barrier, repair mechanism, bioactive constituents, mechanism of action

## Abstract

The skin barrier is essential for maintaining the body’s internal homeostasis, protecting against harmful external substances, and regulating water and electrolyte balance. Traditional Chinese Medicine (TCM) offers notable advantages in restoring skin barrier function due to its diverse components, targets, and pathways. Recent studies have demonstrated that active ingredients in TCM can safely and effectively repair damaged skin barriers, reinstating their proper functions. This review article provides a comprehensive overview of the mechanisms underlying skin barrier damage and explores how the bioactive constituents of TCM contribute to skin barrier repair, thereby offering a theoretical framework to inform clinical practices.

## 1. Introduction

**Figure 1:**
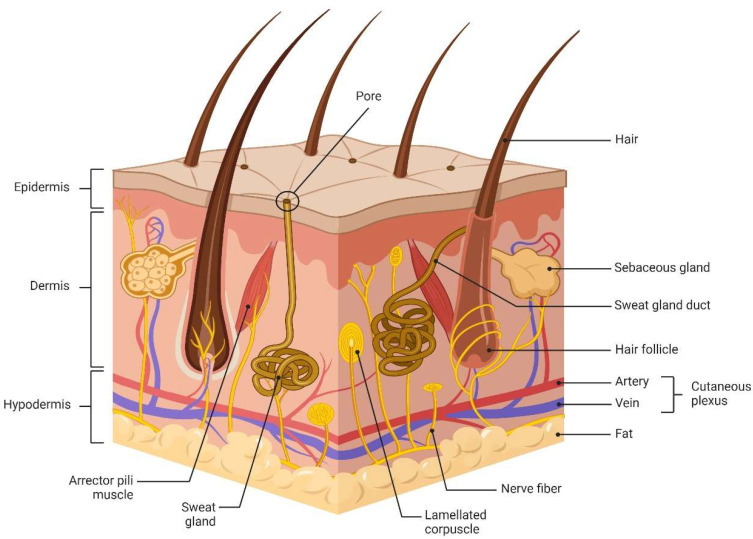
Skin structure.

The skin, being the largest organ in the human body, serves various functions, including acting as a barrier. It is composed of the epidermis, the dermis, and subcutaneous tissue, and it contains accessory organs (sweat glands, sebaceous glands, hair follicles), blood vessels, lymphatic vessels, nerves and muscles, regulating temperature and providing immunity [1]. One of these functions, the skin barrier, is of utmost importance. Serving as the primary defense mechanism of the body, the skin effectively shields against damage caused by external chemical, physical, mechanical, biological, and other factors, while also preventing water and nutrient loss [2]. Figure 1 illustrates the skin’s structure.

Impaired skin barrier function can result in the infiltration of harmful environmental substances into the body, including pathogenic bacteria, allergens, toxins, and irritants, potentially triggering systemic allergic reactions. The prevalence of impaired skin barrier function is on the rise due to shifts in environmental conditions and lifestyle factors, leading to various skin issues such as flushing, papules, scales, itching, and stinging [3]. Many diseases, including eczema, atopic dermatitis, psoriasis, acne, and chloasma, are associated with compromised skin barrier function [4]. Within the European dermatology patient population, male patients make up 48.98%, with female patients slightly outnumbering males at 51.02%. Research indicates that 43.35% of patients have experienced at least one skin disease in the past 12 months. The most prevalent types of skin disease include fungal skin infections, acne, and atopic dermatitis [5].

The treatment of skin diseases in recent years has involved the use of steroids, antihistamines, emollients, and immunomodulators. However, prolonged use of steroids can result in various adverse effects. Prolonged use of steroids can lead to thinning and weakening of the skin, resulting in wrinkles, purple lines, and ecchymoses. Additionally, it may cause hair to become finer and even result in hair loss. Chronic steroid use suppresses immune system function, which increases the risk of skin infections [6]. Furthermore, long-term steroid use may create dependency, and discontinuation can trigger rebound dermatitis [7]. Modern medicine has made significant progress in treating skin diseases through various methods, but different treatments can sometimes have side effects. For example, immunotherapy modulates the immune system to alleviate symptoms or prevent disease progression and has shown remarkable efficacy in treating conditions like melanoma and psoriasis. However, this method can also hyperactivate the immune system, leading to attacks on healthy cells and causing side effects such as fatigue, rashes, and intestinal problems [8]. Isotretinoin has been effective in treating severe acne, but it comes with side effects that should not be overlooked, including potential fetal malformations, dry skin and lips, nosebleeds, and photosensitivity [9]. Additionally, when topical treatments are ineffective, phototherapy is a viable option for controlling symptoms, although it may cause premature skin aging, pigment changes, and an increased risk of skin cancer with long-term use; hence, it is generally not recommended for children [10].

Research has demonstrated that traditional Chinese medicine is both safe and efficacious in the treatment of skin diseases. It has been shown to alleviate itching, decrease skin inflammation, enhance quality of life, reduce reliance on steroids and antibiotics, and prevent recurrence [11].

In order to comprehensively review the mechanism and state-of-the art of traditional Chinese medicine in repairing the skin barrier, a systematic search was conducted on major scientific databases, including Medline, PubMed, ScienceDirect, and Scopus, from 1 January 2013 to 31 November 2023. Manual searches were also conducted to find relevant articles. The literature search process aimed to cover a wide range of studies detailing the mechanisms and therapeutic potential of various TCMs. The specific keywords, ‘skin barrier’ and ‘mechanism of action’, along with their related synonyms and terms, were employed in the search. The criteria for selecting studies in this review were predetermined, and all peer-reviewed research articles, review papers, and clinical trial reports published in English were included. Articles that are not directly related to the pharmacological effects of traditional Chinese medicine and those focused solely on chemical synthesis without involving biological effects were excluded. A total of 149 articles were selected to review in this research.

## 2. Physiological Properties and Functions of Skin Barrier

The skin has a distinct network of immune functions that specialize in recognizing, processing, and presenting antigens. Functionally, the skin barrier can be categorized into four levels, namely the physical barrier, chemical barrier, microbial barrier, and immune barrier. These levels are interconnected and collaborate closely to uphold the skin’s health and defense mechanisms.

Hydrophobic lipids play a crucial role as the skin’s physical barrier, constructing a framework to secure mature keratinocytes. The lipid envelope comprises ceramides, fatty acids, and cholesterol, which interact within the matrix [12]. The alteration of the arrangement and structure of this lipid framework may lead to elevated transepidermal water loss (TEWL) and increased allergen infiltration [13]. Tight junctions (TJs) are integral in forming the side walls of cells, maintaining high solute concentrations between layers to restrict the transport of solutes and TEWL. They also play a crucial role in responding to barrier disruption, with their expression influenced by toll-like receptor (TLR) signaling. Increased TLR signaling can help prevent the entry of external antigens, microorganisms, and other substances into the skin, while also regulating substance transport, proliferation, and differentiation of epidermal cells, as well as lipid secretion [14]. Collectively, these structures and functions serve as the skin’s primary defense mechanism.

The chemical layer of the skin barrier consists of lipids, natural moisturizing factors (NMFs), and compounds that help maintain an acidic pH. These elements are crucial for keeping the skin barrier adequately hydrated, preventing dysbiosis, and optimizing pH for enzyme functions associated with keratinization, desquamation, and lipid synthesis [15]. Lipids in the stratum corneum (SC) protect the skin against TEWL, UV radiation, oxidation, and pathogens. Additionally, lipids also participate in various biological processes, such as biochemical signaling pathways, skin barrier repair, deactivating *Staphylococcus* spp., and shaping skin microbiota. However, lipids also interact with other Gram-positive bacteria to strengthen the physical barrier of the skin, acting as an extracellular ‘mortar’ within the SC [16].

The acid mantle plays a critical role in maintaining the skin’s physiological pH (in the range of 4 to 6), which is essential for homeostasis, fostering antimicrobial conditions, ensuring barrier integrity, and facilitating recovery [17]. Additionally, skin regeneration, desquamation, and lipid metabolism are closely related to skin pH. Proteases such as kallikrein-related peptidase (KLK) and cathepsin mediate skin desquamation by breaking down connections between keratinocytes in the SC [18]. Skin pH affects protease activity and lipid metabolism, thereby influencing the structure of the lipid bilayer in the SC. Ceramide precursors, such as glucosylceramide and sphingomyelin, are initially stored in lamellar bodies found within the stratum granulosum (SG) of the skin [19]. These precursor molecules undergo enzymatic modifications at the junctions between the SG and SC. Specifically, enzymes like β-glucocerebrosidase and acid sphingomyelinase catalyze these modifications to convert the precursors into ceramides. The ceramides are essential components of the intercellular lipids in the outermost layer of the skin [20]. Another important molecule in the skin is profilaggrin, which is primarily composed of L-histidine. The proteolysis of profilaggrin, releasing a significant amount of L-histidine, contributes to the formation of natural moisturizing factors (NMFs) in the SC [21]. Other than L-histidine, NMFs consist of a variety of components essential for maintaining skin hydration and barrier function, including pyrrolidone carboxylic acid, uric acid, lactic acid, urea, citrate, and various sugars. These compounds are naturally present in the skin or can be applied through skincare products like moisturizers to support the skin’s barrier function, maintain hydration and promote overall skin health [22].

Upon analysis of the microbial layer, researchers have discovered a diverse microbiota consisting of symbiotic microorganisms and bacteria that inhabit the outer surface of the dermis [23]. Commensal microorganisms interact with the chemical and immune layers of the skin barrier through signaling mechanisms with host keratinocytes. This interaction prompts the production of antimicrobial peptides (AMPs), such as human beta-defensins (hBDs) and cathelicidins, which are crucial for inhibiting microbial growth on the skin’s surface [15]. Some human beta-defensins, like hBD-1, are constitutively expressed. On the other hand, defensins like hBD-2, hBD-3, and hBD-4 are induced and increased in response to pro-inflammatory cytokines and specific inflammatory conditions, such as psoriasis [15]. Antimicrobial peptides (AMPs) are produced by microorganisms like Staphylococcus epidermidis and Staphylococcus hominis, demonstrating bactericidal properties against the pathogenic Staphylococcus aureus [24]. AMPs play a role in bridging innate and adaptive immune responses by promoting the migration of innate inflammatory cells, enhancing dendritic cells and T cells during infections, and potentially influencing antibody class switching [15]. AMPs may help strengthen the skin barrier by upregulating the proteins that constitute TJs. The presence of commensal microorganisms in the epidermis limits the space and nutrients available for potentially harmful microorganisms. Staphylococcus epidermidis, a healthy commensal organism, plays a role in upregulating TJs proteins and maintaining physical barriers, highlighting the interconnectedness of functional skin layers [25].

In the immune layer of the skin, immune cells play a crucial role in maintaining the stability of the skin barrier. They recognize factors that can disrupt the barrier, communicate with commensal microorganisms, and activate immune pathways. The presence and recruitment of both innate and adaptive cell types ensures that they work together to establish the skin’s immune barrier [26]. Innate immune cells in the skin constantly monitor the external environment using pattern recognition receptors and selectively respond to factors that can breach the barrier. This includes epidermal Langerhans cells (LCs) and dermal dendritic cells (DCs), which play essential roles in detecting and responding to potential threats to the skin barrier. Resident T cells and keratinocytes possess the ability to initiate signaling pathways in response to damage to the skin barrier. Crucial interactions occur between the immune and microbial components of the skin barrier, which work together to maintain a healthy balance, prevent dysbiosis, and promote innate and adaptive immune responses. This interaction also serves to control the growth of commensal microorganisms, ultimately protecting against potentially harmful pathogens, such as *Staphylococcus aureus*. This regulation ensures that these microbial populations do not proliferate to levels that could provoke inflammation [27]. Keratinocytes play a crucial role in maintaining epidermal populations of immune cells through cytokine signaling. Resident memory T cells (Trm) are sustained by cytokinds such as IL-7, IL-15, and TGF-β, while LCs receive support from stimulating factor 1 ligand and TGF-B. Macrophages, on the other hand, rely on IL-34 for their maintenance. Additionally, keratinocytes possesses numerous pattern recognition receptors that can detect both pathogen-associated molecular patterns (PAMPs) and damage-associated molecular patterns (DAMPs) associated with microbial invasion and mechanical stress. This recognition leads to the activation of second messenger pathways such as NF-κB or MAPK, the expression of pro-inflammatory genes, the secretion of cytokines, and the initiation of cell-mediated inflammatory reactions [27].

The skin barrier plays a crucial role in maintaining skin health and overall body defense by preventing moisture loss, resisting harmful external substances, providing physical protection, supporting immune defense, facilitating sensation, and regulating temperature. Understanding the structural and physiological properties of the skin barrier is essential for effectively protecting and repairing it. By adopting healthy lifestyle habits and employing scientifically backed skincare practices, individuals can maintain the integrity of their skin barrier, thereby enhancing overall skin health and ensuring that it remains resilient and functional in its protective role.

## 3. Skin-Related Diseases

**Figure 2:**
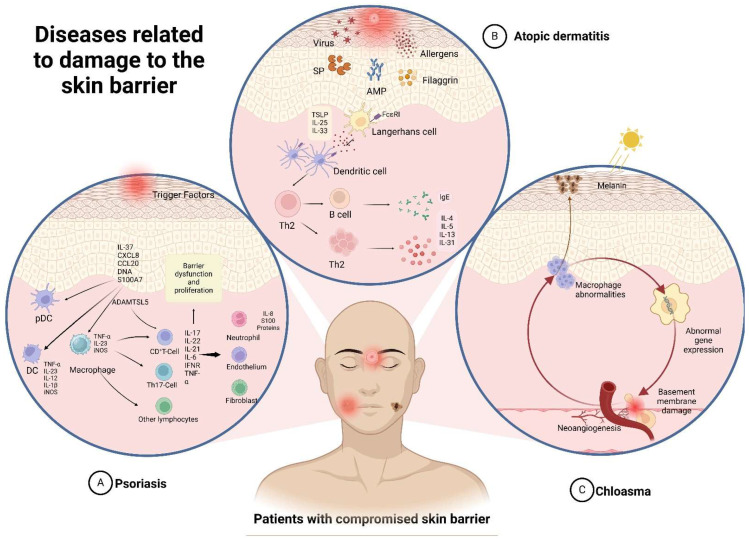
Skin pathogenic mechanism.

Impairment of the skin barrier’s function can lead to various common skin diseases, including atopic dermatitis, psoriasis, eczema, and acne, and may also trigger urticaria and skin cancer. Clinical management typically involves the use of topical steroids, oral antihistamines, or topical moisturizers. However, these skin conditions often recur due to inadequate treatment, repeated allergen exposure, and chronic urticaria inflammation. The following subsection discusses the pathogenesis of several common skin diseases and their typical clinical treatments. Figure 2 illustrates the skin’s pathogenic mechanism.

### 3.1. Atopic Dermatitis

Atopic dermatitis (AD) is a common chronic inflammatory skin disease characterized by eczema, lichenoid lesions, and severe itching. Its pathogenesis is primarily associated with skin barrier dysfunction and immune dysregulation. AD is often associated with asthma, allergies, rhinitis, and other atopic diseases [28]. AD can affect approximately 12% of children and 7.2% of adults, with a higher prevalence observed among young children. Around 60% of children develop the disease before the age of 1, and 90% before the age of 5. The affected areas vary with age, and symptoms include changes in the skin and severe itching, significantly impacting the patient’s quality of life. Research has established a connection between the incidence of AD and keratinocytes, which are influenced by numerous factors. Additional studies have demonstrated that mutations in genes responsible for encoding proteins such as filaggrin and claudin-1 within keratinocytes contribute to the structural fragility of the SC and the increased permeability of TJs. This increased permeability facilitates antigen infiltration, activates dendritic cell (DCs), and triggers the release of thymic stromal lymphopoietin (TSLP). These processes ultimately stimulate a type II adaptive immune response targeting the epidermis. Key cytokines involved in exacerbating barrier dysfunction include IL-4 and IL-13, which suppress filaggrin expression and interfere with TJs. Additionally, IL-4-activated B cells contribute to the production of IgE antibodies against both foreign and self-antigens. TSLP and IgE can trigger itching, perpetuating the disruption of the skin barrier [29]. This breakdown in the skin barrier can result in reduced levels of filaggrin, ceramide, and antimicrobial peptides. Additionally, there can be an increase in serine proteases and serum IgE levels, ultimately leading to immune dysfunction [30].

The prevalence of AD is increasing annually [31]. Various medications, including corticosteroids, topical calcineurin inhibitors, antihistamines, and systemic immunosuppressants, are used for clinical treatment, but these drugs have their limitations. Topical corticosteroids (TCS) and calcineurin inhibitors (TCI) are the primary local treatments for AD and are extensively used in clinical practice. However, prolonged use of TCS on large areas of skin can lead to adverse local and systemic reactions (reference). Abrupt discontinuation of these medications can result in adverse effects, such as sleep disturbances, severe itching, skin pain, and discomfort, due to the complex interactions involving inflammatory cells and cytokines, which can compromise skin integrity [32].

### 3.2. Psoriasis

Psoriasis is a skin disease characterized by chronic inflammatory signaling and the excessive proliferation of keratinocytes. It is characterized by thickening of the epidermis, the abnormal proliferation and differentiation of keratinocytes, and infiltration of inflammatory cells into the skin layers. Psoriasis is characterized by erythema and papules, as well as round patches with silvery white scales as clinical symptoms. Although the exact cause is still unclear, most researchers believe that the abnormal proliferation and apoptosis of keratinocytes are among the main factors contributing to skin damage in this disease. Triggering factors can be classified into internal and external causes [33]. The pathogenesis of psoriasis involves both internal and external factors. Internal causes include genetic predisposition, immune dysregulation, environmental influences, and oxidative stress. External factors primarily involve damage to keratinocytes due to mild trauma, infections, sunburn, systemic medications, stress, physical injury, air pollution, and biological agents.

Psoriasis can occur at any age, but it is common in the elderly. The pathogenesis includes the overexpression of antimicrobial peptides (AMPs) and the activation of plasmacytoid dendritic cells (pDCs), triggered by damaged keratinocytes. Proteins include LL37, β-defensin, and S100 proteins, which are released from damaged cells and contribute to the autoimmune response seen in psoriasis These substances form complexes that activate pDCs to produce type I interferon (IFN), thereby promoting the development of psoriatic plaques. Type I IFN signaling can further enhance the phenotypic maturation of myeloid dendritic cells (mDCs) and participate in the differentiation and function of Th1 and Th17 cells. Additionally, pro-inflammatory dendritic cells release IL-23, which stimulates T cells to produce IL-17. This cascade activates epidermal keratinocytes to release pro-inflammatory cytokines, such as IL-1, IL-6, CXCL1, and CCL20, exacerbating the onset and progression of psoriasis symptoms [34].

Currently, there is a lack of reliable clinical diagnostic methods for psoriasis. Typically, doctors use the Psoriasis Area and Severity Index (PASI) to assess the severity of the disease and determine suitable treatment options. While there is no definitive cure for psoriasis, treatment options vary from topical creams for mild cases to a combination of phototherapy and systemic medications for moderate-to-severe cases. However, these treatments have their limitations and may lead to side effects such as itching, flushing, and high blood pressure with prolonged use [35].

### 3.3. Chloasma

Chloasma is an acquired pigmented skin disease primarily influenced by factors such as sun exposure, hormonal changes, thyroid dysfunction, and so on. These factors lead to abnormal gene expression in exposed skin areas, impacting melanin metabolism, oxidative stress, skin barrier function, and the composition of neural factors [36]. Chloasma is common in young and middle-aged Asian women. It manifests as bilaterally symmetrical brown spots of varying shades, significantly affecting appearance and increasing psychological stress for patients. Further research has found that the formation of chloasma involves several factors, including excessive melanin production, an increased number of melanocytes and mast cells, abnormal gene regulation, neovascularization, and damage to the basement membrane. Ultraviolet (UV) rays play a significant role in promoting melanin production in the skin. Studies have shown that melanin production is regulated through various signal transduction pathways. Examples include Wnt/β-catenin, PI3K/Akt, cAMP/PKA, and SCF/c-kit-mediated signaling pathways. UV irradiation leads to the expression of several melanocyte-specific genes and stimulates the release of key factors involved in melanin synthesis. This results in a significant increase in melanocyte-specific gene expression, thereby causing melanin synthesis. Melanin is significantly increased in the affected skin layers, which may be caused by abnormal cell–cell interactions. Furthermore, the pathogenesis of chloasma is known to be associated with inflammatory mediators, oxidative stress, neuroactive molecules, and sebocytes [37].

Because of its complex pathogenesis, chloasma treatment is difficult and can easily lead to recurrence, seriously affecting the patient’s life quality. Effective management of chloasma requires long-term treatment, and current treatment methods include the topical application of various substances, chemical peels, and laser therapy. Traditional treatment methods primarily involve laser elimination or the regeneration of fibroblasts to improve the skin environment. However, studies have shown that laser treatment can easily cause recurrence and damage facial collagen, leading to patient dissatisfaction [38] Topical drug options for chloasma include hydroquinone, retinoic acid, and glucocorticoids, among others. However, these treatments can be painful and have side effects, making them difficult for patients to tolerate. Consequently, there is an urgent need for new drug treatments that can improve the physiological and psychological conditions of patients with chloasma.

### 3.4. Other Related Diseases

Eczema is a common allergic inflammatory skin disease, mainly characterized by skin itching, scales, erythema, and exudation [39]. Approximately 20% of children and 1–3% of adults worldwide suffer from eczema [40]. In recent years, the incidence of eczema has been increasing, with complex and diverse causes. The condition is persistent and prone to recurrence. Severe cases can cause intense itching or cracking pain, significantly impacting patients’ daily life, work, and physical and mental health. The pathogenesis of eczema involves genetic and environmental factors, with immune cells and cytokines, such as TH2 (IL-4, IL-5, IL-13, and IL-31), TH17 (IL-17A, IL-17F, IL-22, and IL-26), and TH9 cells, playing crucial roles in the disease’s development [41]. In terms of treatment, modern medicine utilizes topical glucocorticoids, calcineurin inhibitors, glucocorticoids, antihistamines, immunosuppressants, and other drugs to manage eczema. Topical corticosteroids are often the first choices. However, long-term use can lead to drug resistance and poor patient compliance, necessitating combined systemic therapy. Abrupt discontinuation of steroids may cause adverse reactions, so treatment should be administered with caution [42].

Acne is a chronic inflammatory skin disease affecting the pilosebaceous glands. Its primary clinical features include various lesions on the face, chest and back, such as comedones, papules, pustules, nodules, cysts and scars. The condition typically manifests during adolescence and can persist for years. Symptoms often improve or resolve after adolescence, with non-inflammatory or inflammatory acne commonly appearing on the face, neck, trunk, and back [43]. Acne activates innate immunity via the expression of protease activated receptors (PARs), tumor necrosis factor (TNF) α and toll-like receptors (TLRs), and the production of interferon (INF) γ, interleukins (IL-8, IL12, IL-1), TNF, and matrix metalloproteinases (MMPs) by keratinocytes, resulting in the hyperkeratinization of the pilosebaceous unit [44]. Current medical treatments for acne primarily involve topical benzoyl peroxide, antibiotics, or retinoic acid, as well as oral medications like minocycline and zinc sulfate. While these treatments can achieve certain clinical effects, prolonged use may lead to the development of drug-resistant strains of bacteria, such as increased levels of Propionibacterium acnes, elevated blood lipid levels, and other side effects. Acne is also prone to recurrence if not promptly and effectively treated, potentially leading to scarring and psychological issues that impact patients’ life quality [45].

Mast cells are the primary effector cells in urticaria. These cells are widely distributed in the skin, mucosa, and other areas of the body, and they have high-affinity immunoglobulin E (IgE) receptors. Mast-cell degranulation leads to the rapid release of various inflammatory mediators, such as histamine, leukotrienes, and prostaglandins, which, in turn, cause vasodilation and leakage of plasma in and below the skin. There is also a more delayed (4–8 h) secretion of inflammatory cytokines (e.g., tumor necrosis factor, interleukin 4 and 5) that potentially leads to further inflammatory responses and longer-lasting lesions [46].

DNA damage serves as a critical link in the development of skin cancer. When the skin barrier is compromised, carcinogens are more likely to penetrate skin cells and directly impact the cell’s DNA. This interaction can trigger mutations in the genetic material of the cells, leading to a loss of control over the normal processes of cell growth and differentiation [47]. Research indicates that exposure to harmful external substances and carcinogens can elicit an inflammatory response in the skin. Prolonged chronic inflammation may result in abnormal cell proliferation and an imbalance in apoptosis, thereby heightening the risk of skin cancer [48]. Additionally, research has demonstrated that the immune system is crucial in eliminating abnormal cells and preventing tumor development. When the skin barrier is compromised, the functionality of immune cells may be impaired, allowing abnormal cells to evade detection by the immune system, thereby increasing the risk of skin cancer [49]. In summary, damage to the skin barrier makes it easier for carcinogens to enter the skin, and increases the risk of skin cancer through multiple mechanisms such as causing DNA damage, promoting chronic inflammation, and weakening immune function. Therefore, maintaining the integrity and health of the skin barrier is of vital importance in preventing the occurrence of skin cancer.

## 4. The Mechanism and Targets of Traditional Chinese Medicine in Restoring Skin Barrier

In recent years, traditional Chinese medicine (TCM) has assumed a crucial role in treating skin diseases. It is noted for its significant therapeutic effects and minimal side effects, positioning it as an alternative therapy for managing and controlling various skin conditions. There is a growing demand for natural plant-based products, further boosting TCM’s popularity in dermatological treatments. Recent studies have demonstrated that the bioactive components of TCM effectively repair the skin barrier. These findings not only establish a theoretical basis for, but also highlight the practical value of using TCM in clinical treatments for skin diseases.

**Figure 3:**
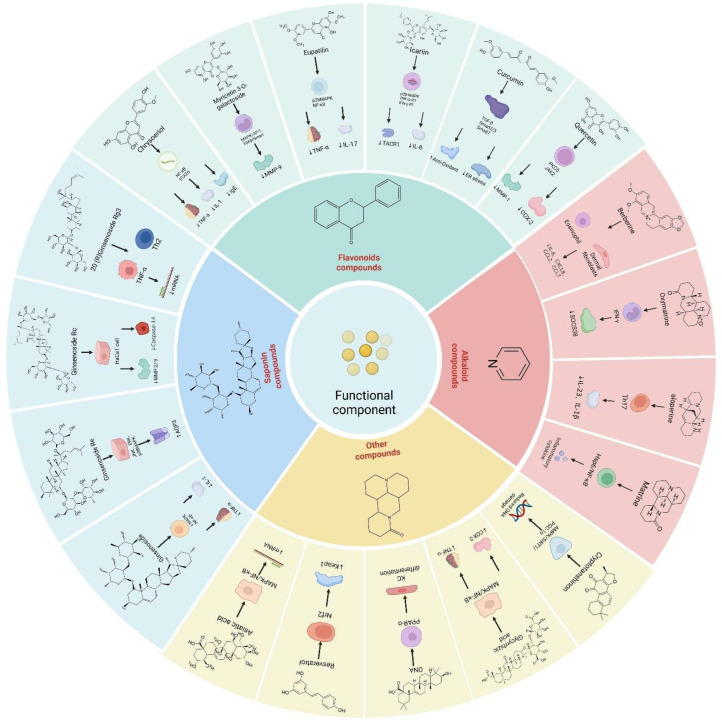
Mechanisms of action of functional factors.

The mechanism of action of the bioactive constituents (functional factors [50]) of TCM in repairing the skin barrier is intricate and involves multiple signaling pathways. External stimuli can activate several signaling pathways, including the p38 protein kinase, NF-κB, MAPK, Keap1-Nrf2-ARE, Nrf2/ARE, and TRPV1 [51]. The activation of these pathways triggers a cascade of biological reactions, such as caspase-3 activation, oxidative stress, the abnormal expression of MMPs, inflammatory responses, DNA damage, autophagy, and excessive melanin synthesis. The bioactive constituents of TCM can enhance skin barrier function and promote repair by modulating these mechanisms. Figure 3 shows the mechanisms of action of functional factors.

### 4.1. Ginsenoside

Ginsenosides, extracted from the roots, stems, and leaves of ginseng, are recognized as pharmacologically active compounds that contribute significantly to skin barrier repair. They exhibit various beneficial properties, including immune enhancement, metabolic boosting, anti-tumor effects, fatigue reduction, and anti-aging properties [52]. Ginsenosides are widely utilized for their protective and therapeutic effects on various systems, including the cardiovascular, nervous, immune, and endocrine systems. Additionally, studies have highlighted their diverse biological functions, such as anti-inflammatory and anti-cancer properties [53].

Further research has demonstrated that ginsenosides have significant effects on the treatment of AD. Kim et al. showed that ginsenoside Rh2 and Rg3 can reduce the increase in TNF-α and IL-4 mRNA expression induced by TNCB, exerting anti-inflammatory effects and inhibiting TNF-α mRNA expression in vivo, thus effectively treating AD in mice [54]. Additionally, Kee et al. conducted a study using mouse models of anaphylactic shock and AD-like skin lesions to evaluate the anti-allergic effects of Korean red ginseng. Their findings indicated that the aqueous extract of Korean red ginseng was effective in reducing the production of pro-inflammatory cytokines and inhibiting the release of TNF-α [55].

Sohn et al. conducted an experiment using DNCB to induce AD-like skin lesions in Balb/c mice, monitoring their scratching behavior and measuring the levels of IL-4, IL-10, serum IgE, and splenocytes through reverse-transcription methods. They utilized various techniques, such as polymerase chain reaction, Western blotting, and ELISA to assess Korean red ginseng’s effects on DNCB-induced MAPKs and Ikaros. The study demonstrated that topical Korean red ginseng administration significantly improved AD symptoms and reduced scratching behaviors in mice. The Korean red ginseng exhibited notable effects in the mouse model of Alzheimer’s Disease (AD) induced by DNcB. Specifically, in these AD-like mice, the topical application of RG led to significant improvements in the skin lesions associated with the condition. Furthermore, RG also resulted in a reduction of cytokines activated by Th2 cells, including IL-4 and IL-10, along with a decrease in serum IgE levels. The anti-atopic effects of RG appear to be primarily mediated through the modulation of specific signaling pathways. In particular, RG seems to inhibit the MAPK signaling cascades, which encompass ERK1/2, JNK, and p38 MAPK. Concurrently, RG activates CK2α, which in turn plays a role in further reducing the transcriptional activity of Ikaros. This inhibition of Ikaros by RG seems to contribute to a downregulation of IL-4 and IL-10 expression in splenocytes [56].

Numerous studies on skin anti-aging demonstrate that ginsenosides play a crucial role in effectively treating skin barrier damage induced by ultraviolet radiation. Oh et al. conducted a detailed study investigating the skin-protecting effects of ginsenoside Rc against UVB-induced damage using HaCaT cells. Their research highlighted ginsenoside Rc’s potential in counteracting photoaging and preserving skin barrier function. The results indicated that ginsenoside Rc effectively inhibited the increase in ROS production and MMP-2/-9 levels in UVB-exposed HaCaT keratinocytes. Additionally, the ginsenoside Rc maintained GSH content and SOD activity; GSH and SOD are crucial antioxidants in skin cells. Moreover, the ginsenoside Rc promoted caspase-14 activity and prevented the downregulation of filaggrin expression, thereby supporting its role in protecting against UVB-induced skin damage and aging [57]^.^

Oh et al. assessed the skin anti-photoaging properties of ginsenoside Rb1 in human dermal HaCaT keratinocytes. Their findings demonstrate that ginsenoside Rb1 boosts the antioxidant capacity of keratinocytes by neutralizing ROS and decreasing MMP-2 levels. These effects contribute to the anti-aging benefits of ginsenoside Rb 1, highlighting its potential in protecting skin cells against photoaging [58].

Li et al. employed the UVB-irradiated BALB/c hairless mouse model to assess the efficacy of ginsenoside in preserving skin epidermal thickness and reducing TEWL. They also investigated the impact of ginsenoside on filaggrin (FLG) degradation, skin barrier function as indicated by involucrin (IVL) protein levels, claudin-1 (Cldn-1) expression, aquaporin 3 (AQP3) levels, and MAPK phosphorylation. The findings indicated that the ginsenoside enhanced epidermal barrier function after damage by UVB and reinstated the levels of protein expression and distribution for FLG, IVL, Cldn-1, and AQP3 in the epidermis. Additional research revealed that ginsenoside suppressed JNK in HaCaT cells irradiated with UVB, as well as ERK phosphorylation and the p38MAPK pathway, resulting in the increased expression of IVL and AQP3 [51].

In a study by Liu et al., the potential of ginsenoside C-Mx in protecting human dermal fibroblasts (NHDF) from UVB-induced damage was explored. The findings indicated that the ginsenoside C-Mx demonstrated the ability to mitigate the intracellular expression of ROS, MMP-1, and IL-6 induced by UVB while promoting the secretion of TGF-β and type I procollagen. Additionally, the ginsenoside C-Mx was able to counteract the UVB-induced decrease in type I procollagen by modulating the TGF-β/Smad signaling pathway. Furthermore, it was observed that the ginsenoside C-Mx inhibited the activation of the MMP inducer AP-1 transcription factor. This compound also displayed significant antioxidant properties by enhancing the nuclear accumulation of Nrf2, resulting in the increased expression of cytoprotective antioxidants like HO-1 and NQO-1 [59].

Overall, the study revealed that ginsenoside C-Mx holds promise as a protective agent against UVB-induced damage in human dermal fibroblasts. Its ability to regulate key pathways, such as TGF-β/Smad signaling, and inhibit the activation of MMP inducer AP-1 transcription factor demonstrates its potential in restoring cellular health and promoting collagen production. Additionally, the antioxidant capacity of ginsenoside C-Mx, as evidenced by its impact on the expression of cytoprotective antioxidants, further highlights its beneficial effects in combating oxidative stress and maintaining cellular integrity. This comprehensive protective mechanism underscores the therapeutic potential of ginsenoside C-Mx in skin-care and anti-aging applications.

In summary, ginsenosides can exert their skin-protecting properties by inhibiting multiple signaling pathways, such as JNK, ERK phosphorylation, p38MAPK, and NF-κB. By modulating these pathways, ginsenoside contributes to the improvement and repair of the skin barrier. This provides new directions for treating various skin diseases, offering potential therapeutic benefits in enhancing skin health and mitigating damage. Table 1 lists the ginsenosides’ mechanisms of action.

### 4.2. Flavonoids

Natural flavonoids are commonly found in plants as either O-glycosides or C-glycosides. Research indicates that these compounds are crucial for various biological functions and offer numerous health benefits, such as reducing inflammation, combating oxidative stress, preventing mutations, inhibiting cancer growth, and fighting off bacteria. They have the capacity to shield cell membranes, reduce cholesterol levels, combat atherosclerosis and cancer, alleviate spasms, and function as antioxidants, inhibitors of nitric oxide synthase, and agents for photoprotection [64]. These multifaceted properties underscore the therapeutic potential of flavonoids in promoting overall health and preventing a wide range of diseases.

Flavonoids exhibit targeted effects in repairing the skin barrier. Studies have found that aureiodictyin reduces the protein levels of phosphorylated p65 (Ser536), phosphorylated STAT3 (Tyr705), inducible iNOS, COX-2, IL-6, IL-1β, and TNF-α in the swollen ears of mice. In vitro experiments also demonstrated its ability to decrease the production of NO and prostaglandin E2 by cells, inhibit the phosphorylation of κB (Ser32), p65 (Ser536), and Janus kinase 2 (Tyr1007/1008), reduce the nuclear localization of p50, p65, and STAT3, and lower the mRNA levels of the pro-inflammatory cytokines IL-1, IL-3β, and TNF-α, which are transcriptionally regulated by NF-κB and STAT6 in cell models [65].

Sangaraju et al. studied the effect of galangin (GAL) on IMQ-induced psoriasis-like skin inflammation. The GAL significantly reduced the IMQ-induced PASI score, as well as skin and ear thickness, hematological markers, and nitrite levels. It also regulated the protein levels of pro-inflammatory mediators COX-2 and iNOS, the NF-κB pathway, and pro-inflammatory cytokines IL-17, IL-23, IL-1β, and IL-6 in the skin. Additionally, compared to the IMQ group, the GAL restored the levels of antioxidant markers such as SOD, CAT, GST, GSH, GR, and Vit-C, the anti-inflammatory cytokine IL-10, and the protein Nrf2/HO-1 in the skin [66].

Flavonoids demonstrate therapeutic effects on various skin diseases. Luteolin 7-O-glucoside has been shown to possess anti-inflammatory effects in AD [67]. Research indicated that luteolin 7-O-glucoside reduces serum IgE and IL-4 levels, increases skin hydration, and exhibits strong anti-atopic dermatitis activity. Bai et al. investigated the effects of isoflavins from mugwort leaves on psoriasis using HaCaT cells and an IMQ-induced mouse model. In vitro experiments revealed that isoflavins inhibited the p38-MAPK and NF-κB signaling pathways, thereby reducing the excessive proliferation of HaCaT cells stimulated by LPS. In vivo studies showed that isozoranthin reduced TNF-α, IL-6, IL-23, and IL-17 levels in the sera of mice, effectively alleviating IMQ-induced psoriasis in mice [68]. Liu et al. utilized IMQ or TNF-α to induce psoriasis-like models in mice or HaCaT cells to study the effects of cimiculin. The results demonstrated that cimiculin reduced epidermal hyperplsia, PASI scores, ear thickness, and histological psoriasis-like lesions in mice. Cimiculin also lowered levels of GSH, SOD, and CAT. Mechanistically, cimiculin inhibited the upregulation of pro-inflammatory cytokines, including TNF-α, IL-6, IL-1β, IL-17A, and IL-22. It achieved this by inhibiting the phosphorylation of NF-κB (IκB and p65) and MAPK (JNK, ERK and p38) signaling pathways activated by IMQ. Additionally, cimiculin induced the downregulation of ICAM-1 and inhibited inflammatory factors in TNF-α-treated cells [69].

Xiong H et al. used a TNF-α-induced HaCaT cell inflammation model and an IMQ-induced psoriasis animal model to study the effects of glycyrrhizic acid (GL) on the skin. In vitro experiments showed that the GL reduced the level of ICAM-1 in TNF-α-stimulated HaCaT cells, inhibited monocyte adhesion to keratinocytes, suppressed the phosphorylation of p65 after IκB degradation, and blocked ERK and the phosphorylation of p38-MAPK. In vivo experiments demonstrated that GL delayed the onset of psoriasis in mice, thereby reducing ICAM-1 expression in epidermal tissue [70].

Kong et al. studied how icariin inhibited the inflammatory response induced by TNF-α/IFN-γ through the p38-MAPK signaling pathway in human keratinocytes. The results showed that icariin inhibited the production of IL-6, IL-8, IL-1β, and MCP-1 induced by TNF-α/IFN-γ. Additionally, icariin reduced IL-8 and IL-1β in HaCaT cells, as well as the expression of ICAM-1 and TACR1 genes, indicating that icariin mediated these effects by inhibiting the p38-MAPK signaling pathway and regulating TNF-α-R1 and IFN-γ-R1 signals [71]. Table 2 lists flavonoids’ mechanisms of action.

### 4.3. Alkaloid

In traditional Chinese medicine, alkaloids constitute a category of naturally occurring organic chemicals commonly found in a variety of Chinese herbal remedies. They possess complex structures and exhibit a wide array of biological activities, including anti-tumor, anti-viral, anti-inflammatory, antibacterial, analgesic, and immune regulatory effects. Alkaloids are employed in medicine as local anesthetics, stimulants, analgesics, anticancer drugs, antihypertensive agents, and antiarrhythmic medications [86].

Studies have demonstrated significant the therapeutic effects of traditional Chinse medicine alkaloids on skin-related diseases. Gao et al. used TNF-α and IFN-γ to stimulate HaCaT cells and treated them with oxymatrine. The results showed that the oxymatrine sensitized the HaCaT cells to the IFN-γ pathway and repaired the skin barrier by activating p1, JNK, and Akt, while downregulating MDC, ICAM-1, and SOCS1 [87]. Chan et al. investigated the pathological changes in psoriasis-like inflammation induced by the transdermal delivery of capsaicin. The authors used an imiquimod-induced psoriasis-like mouse model to evaluate the therapeutic effect of the topical application of capsaicin. The results demonstrated that capsaicin administration inhibited the imiquimod-induced activation of IL-23/IL-17 pathways. Psoriasis-like erythematous appearance and microscopic features were significantly reduced, along with a notable decrease in the tissue gene expression of core psoriasis cytokines, such as IL-23, IL-17A, IL-22, TNF-α, and IL-6, after the capsaicin treatment [88].

Huang et al. explored the pharmacological effects and mechanisms of matrine on AD. The results showed that matrine reduces the expression of heat shock protein 90 (Hsp90) and decreases the levels of Th2 cytokines in ear tissue and serum. Additionally, it inhibits the Hsp6/NF-κB signaling axis in HaCaT cells, thereby suppressing the secretion of inflammatory cytokines. These findings suggest that matrine can modulate Th2/Th90 inflammatory responses and potentially alleviate skin-related diseases [89].

Tsang et al. investigated the anti-inflammatory effect of berberine in AD-like skin inflammation. The authors found that berberine effectively inhibits the release of IL-6 in eosinophil culture and eosinophil–dermal fibroblast co-culture, as well as the release of CXCL8, CCL2, and CCL7. This action helps to improve allergic inflammation and mitigate the activation state of eosinophils [90].

Norisoboldine, an isoquinoline alkaloid from Wuyao, was investigated for its impact on NFAT activation and its potential in treating AD. The study utilized a luciferase gene assay, K562-luc cells, and Western blotting to examine NFAT dephosphorylation in K562-luc and Jurkat cells. Additionally, real-time fluorescence quantitative PCR was employed to detect IL-2 expression in Jurkat cells. The results indicated that norisoboldine inhibited IL-2 expression in Jurkat cells induced by PMA plus ionomycin, and reduced mRNA levels of INF-γ, TNF-α, IL-4, and IL-6 in mouse ears [91].

Zhou et al. investigated the therapeutic effect of squamine on psoriasis using flow cytometry, Western blot analysis, and real-time fluorescence quantitative PCR for analysis. The findings revealed that the squamine not only inhibited Th17 differentiation, but also suppressed dendritic cell activation, thereby reducing the expression and secretion of pro-inflammatory cytokines, particularly IL-23 and IL-1β [92].

Alkaloid compounds have demonstrated extensive potential in repairing the skin barrier. They achieve this by inhibiting various proteins and inflammatory factors through mechanisms such as IFN-γ pathway inhibition, Hsp6/NF-κB signaling suppression, the modulation of Th2 and Th17 differentiation, and reductions in inflammatory factor mRNA expression. Notably, alkaloids inhibit IL-23, IL-1β, and IL-6, thereby exerting anti-inflammatory and antioxidant effects, promoting cell proliferation and differentiation, and enhancing skin barrier function. These compounds hold promise for treating damaged skin barriers. Future research should delve deeper into their specific molecular mechanisms and explore their clinical applications to offer more treatment options for skin barrier dysfunction. Table 3 summarizes alkaloids’ mechanisms of action.

### 4.4. Carbohydrates

Polysaccharides are polar complex macromolecular compounds composed of monosaccharides linked by glycosidic bonds, with a degree of polymerization exceeding 10. Polysaccharide drugs possess intricate molecular structures, perform diverse biological functions, and interact with multiple molecular targets [94]. Consequently, they play crucial roles in Chinese herbal medicine, in which various polysaccharides exhibit activities such as anti-tumor, antioxidant, anti-diabetic, anti-radiation, anti-viral, hypolipidemic, and immunomodulatory effects [95]. As a result, polysaccharides have garnered significant attention in both scientific research and traditional medicine practices.

Carbohydrate compounds play a significant role in repairing the skin barrier. Li et al. studied the effects of ginseng oligosaccharide extract (GSO), demonstrating its ability to reduce UVB-induced epidermal thickening and water loss. Additionally, GSO improved levels of FLG, IVL, and AQP3 proteins. Further investigation revealed that mRNA and related proteins associated with desquamation, such as SPINK5, KLK5, KLK7, and DSG1, returned to normal levels [96].

Li et al. investigated the photoprotective effects of Lycium barbarum polysaccharide (LBP) on UVB-induced photodamage in HaCaT cells. The findings indicated that LBP reduced cell viability, ROS production, and mitigated DNA damage. Moreover, LBP inhibited p38 MAPK activation, reversed caspase-3 activation, and suppressed MMP-9 expression. LBP promoted Nrf2 nuclear translocation and increased the expression of Nrf2-dependent ARE target genes [97].

Chen et al. explored the effects and mechanisms of astragalus polysaccharide (APS) in improving imiquimod-induced psoriasis in mice. The authors measured inflammatory factor secretion using ELISA and skin macrophage infiltration by flow cytometry to assess the APS’s impact on psoriasis. The results demonstrated that high-dose APS significantly reduced skin-tissue macrophage infiltration. APS improved psoriasis-like dermatitis in mice by inhibiting skin macrophage infiltration and reducing serum levels of TNF-α, IL-1β and IL-6 [98].

Yuan et al. utilized BALB/c female mice as animal models and nerve growth factor (NFG)-activated PC12 cells as cutaneous nerve cell models to investigate the repairing effects of aloe polysaccharide (AP) on UVB-damaged nerve cells. The authors employed an MTT assay for cell viability analysis, TUNEL and annexin-V/PI staining for cell apoptosis detection, flow cytometry (FCM) for cell-cycle analysis, an enzyme-linked immunosorbent assay (ELISA) for oxidative stress and antioxidant capacity assessment, and real-time fluorescence quantitative PCR plus Western blotting for detecting levels of Bax, Bcl-2, Caspase-3, Cyclin D1, Keap1, Nrf2, GCLC, and GSTP1 expression. The results demonstrated that AP inhibited cell apoptosis, enhanced cell viability, and improved antioxidant capacity in UVB-damaged never cells. Furthermore, AP upregulated the expression levels of Keap1, Nrf2, GCLC, and GSTP1, indicating the activation of the Keap1/Nrf2/ARE signaling pathway. These findings suggest that AP not only repairs UVB-induced damage in nerve cells, but also ameliorates UVB-induced damage in NFG-activated skin nerve cells through the Keap1/Nrf2/ARE pathway [99].

In summary, polysaccharides exhibit potential in repairing skin barrier damage by enhancing cellular functions and modulating signaling pathways such as Keap1/Nrf2/ARE and MAPK. This capability extends to improving skin diseases associated with barrier dysfunction, laying a foundation for future research in this area of dermatology. Table 4 summarizes carbohydrates’ mechanisms of action.

### 4.5. Other Compounds

Other compounds, such as coumarins, phenolic acids, and pentacyclic triterpenoids, also play a role in repairing the skin barrier.

The effects of gallic acid following topical skin application were investigated by monitoring transepidermal water loss, erythema index, and protein expression [100]. The study’s results indicated that the expression levels of reactive oxygen species (ROS), interleukin-6, and matrix metalloproteinase-1 (MMP-1) were significantly inhibited in skin treated with gallic acid. This effect effectively reduces skin dryness, thickness, and wrinkle formation by negatively regulating the secretion of MMP-1 while positively regulating the expression of elastin, type I procollagen, and transforming growth factor-β1. After exploring the protective effects and potential mechanisms of GA on psoriasis-like skin diseases in vitro and in vivo [101], the results showed that GA can significantly reduce the mRNA and protein expression levels of psoriasis-related keratin 16 and keratin 17. In addition, GA significantly improved the skin lesion area and severity scores of psoriasis-like mice, while also significantly reducing epidermal hyperplasia in the mice. The study also found that GA inhibited Nrf2 activity in the process of targeting keratin 16 and keratin 17. The study investigated the effect of GA on the inflammatory response induced by DNCB. The experiments involved measuring the thicknesses of mouse ears and conducting histopathological examinations. Additionally, changes in serum levels of IgE and TNF-α were analyzed to elucidate the mechanism of action of GA. The mRNA expression levels of TNF-α, IL-4, IFN-γ, and IL-17 were assessed to understand the impact of GA on lymph nodes. The study also examined the influence of GA on regulatory T cells (Treg) and TH17 cells. The results demonstrated that the GA significantly reduced the thicknesses of the mouse ears. Compared to the model group, the serum IgE and TNF-α levels were markedly lower in the GA group. Furthermore, the lymph node weight, as well as the TNF-α levels in the lymph nodes of the mice treated with the GA, showed significant reductions. The expression of IL-4, IFN-γ, and IL-17 mRNA was also significantly decreased. In comparison to the model group, the expressions of IL-4, IL-5, IL-17, and IL-23 were reduced, while the expressions of IL-10 and TGF-β were significantly increased. The analysis of the Th17 cell signature genes revealed that the ROR-γt expression was significantly lower in the GA group, whereas the SOCS3 expression was elevated. These findings suggest that GA exerts a therapeutic effect on DNCB-induced atopic dermatitis (AD) inflammation [102].

Oleanolic acid, a common pentacyclic triterpenoid found widely in plants in its free acid form, exhibits various pharmacological activities, including hepatoprotective, anti-inflammatory, antioxidant, and anticancer properties [103]. Studies have indicated that oleanolic acid and its derivatives can enhance the recovery of the mouse epidermal permeability barrier. For instance, experiments involving tape-stripped mouse skin assessed parameters such as TEWL, hydration levels, and morphological changes using electron microscopy. The findings revealed improvements in TWEL stability, the increased presence of secretions and lamellar bodies, and the notable formation of lipid bilayers. Moreover, in HaCaT cells treated with ursolic acid (UA) and oleanolic acid (ONA), protein expression levels of PPAR-α, involucrin, loricrin, and filaggrin were significantly enhanced, be twofold and threefold, respectively, indicating that these compounds promote epidermal keratinocyte differentiation and contribute to skin barrier function recovery through the PPAR-α pathway [104].

Tsang et al. explored the anti-inflammatory effects of gallic acid and chlorogenic acid in AD-like skin inflammation. The experiments demonstrated that these compounds effectively inhibit the release of pro-inflammatory cytokine IL-6 in IL-31- and IL-33-treated eosinophil–dermal fibroblast co-cultures, as well as the release of chemokines CCL7 and CXCL8 [90].

In a study conducted by Kim et al., the therapeutic effects and anti-inflammatory mechanisms of *Terminalia chebula* extract, a traditional Chinese medicine, were investigated in an in vivo AD mouse model. The findings revealed that the extract decreased serum levels of IgE, histamine, and inflammation-related mediators, such as MDC, TARC, RANTES, and TSLP. Additionally, the extract strongly inhibited the expression of inflammatory chemokines RANTES and MDC in HaCaT cells stimulated with IFN-γ/TNF-α. This inhibition was associated with the suppression of phosphorylated STAT1/3 and NK-κB subunits, as well as the nuclear translocation of NF-κB. Furthermore, the extract effectively suppressed the transcription of IFNγ, IL-6, IL-8, and MCP-1 in IFNγ/TNF-α-stimulated HaCaT cells, demonstrating its potential for treating AD [105].

Similarly, Song et al. studied the therapeutic effect of galangal extract on AD mice. The results showed that the extract inhibited the expression of pro-inflammatory factors such as MDC, RANTES, IP-10, and I-TAC in HaCaT cells stimulated by IFN-γ and TNF-α. It also inhibited the phosphorylation of MAPK, NF-κB, and STAT1. These findings suggest that these Chinese herbal extracts may have significant anti-inflammatory and therapeutic effects on AD [106].

Ming et al. pointed out that the inhibitory effect of Daqing leaf extract extends to AD mice and HaCaT cells at a mechanistic level. Their findings indicate that the extract reduces the expression levels of extracellular-signal-regulated kinase and p38-mitogen-activated protein kinase proteins. Moreover, it hinders the translocation of p65 from the cytoplasm to the nucleus, ultimately leading to the decreased mRNA expression of TNF-α, IFN-γ, IL-6, and IL-13 in skin tissues affected by disease. Furthermore, in HaCaT cells, the extract demonstrated the ability to impede the regulation of activated T cells and the production of TARC, MDC, MCP-1, and MIP-3a [107]. Han et al. discovered that extracts from *Artemisia annua* (AWE) significantly impacted a mouse model of DNCB-induced AD. The findings indicated that the AWE alleviated AD symptoms in the mice and suppressed the mRNA and protein expressions of IgE, IL-4, IL-6, IL-13, IL-17, TNF-α, and TSLP. Moreover, the AWE treatment also reduced the phosphorylation levels of p38 MAPK and NFκB in the ear tissues of the AD mice [108].

To summarize, the repair of the damaged skin barrier is facilitated by coumarins, phenolic acids, and pentacyclic triterpenoids through the activation of signaling pathways such as ITK, PLC-γ1, NF-κB, and MAPK. These compounds have significant effects, providing a basis and new ideas for their future clinical application in skin diseases. Table 5 lists the compounds’ mechanisms of action.

## 5. State-of-the-Art Research on Traditional Chinese Medicine

With the advancement of modern pharmacology and clinical trials, increasing numbers of studies have reported the application of active ingredients and preparations from traditional Chinese medicine in repairing the skin barrier. Researchers have begun to develop traditional Chinese medicines with skin barrier functions to treat skin diseases such as psoriasis, AD, and acne. Therefore, traditional Chinese medicine has great development potential for repairing the skin barrier, providing broad prospects for its application in the treatment of skin diseases.

Kim et al. conducted an 8-week open, non-comparative clinical study using red ginseng extract to treat patients with AD. The research results showed that red ginseng extract has significant therapeutic effects on patients with AD. It can significantly reduce eczema area and severity index scores, improve itching and sleep disturbance, and reduce the use of topical medications [124]. Lee et al. conducted an 18-week clinical trial on AD patients using red ginseng extract and verified the clinical efficacy of the extract through the SCORAD index, as well as TEWL, DI, SI, and the skin-surface moisture rate. All the indicators improved after 16 weeks, showing that the extract could improve skin barrier function and reduce serum IgE levels without specific side effects. These findings suggest that the extract may have potentially beneficial effects in improving disease severity and skin barrier function, and in alleviating itching and sleep disturbance [125].

Kim et al. studied AD-like skin lesions in an NC/Nga mouse model induced using DNFB. The results showed that Uncaria could inhibit the development of AD in this model by reducing the production of IFN-γ [126]. Theoharides et al. treated four patients with AD and psoriasis with tetramethoxyluteolin. All the patients experienced improvements in their skin conditions, demonstrating the potential role of this ingredient in improving different skin conditions [127].

Lee et al. extracted an alkaloid-rich component, INM-A, from Indigo Naturalis and analyzed its chemical characteristics and anti-psoriasis activity to determine its in vitro mechanism and in vivo efficacy for psoriasis treatment. The results showed that INM-A can significantly improve the skin condition of mice with psoriasis, reduce the levels of IL-17A, and inhibit polarized Th17 cells. Additionally, INM-A targets IL-17A, which can inhibit inflammation and oxidative stress caused by OXPHOS in skin cells [128].

Shi et al. studied the therapeutic effect of oxymatrine on patients with severe plaque psoriasis. The authors stained skin tissue with proliferating cell nuclear antigen (PCNA), Ki-67, and Bcl-2, and identified cells using terminal deoxynucleotidyl-transferase-mediated dUTP nick labeling (TUNEL). The results showed that oxymatrine regulated mitosis, inhibited the overexpression of PCNA and Ki-67 in skin lesions, and promoted the recovery of apoptotic Bcl-2 expression, thereby improving psoriasis skin lesions [129]. Zhou et al. conducted a clinical trial of p-oxymatrine in the treatment of patients with relapsed severe plaque psoriasis. The findings indicated that the oxymatrine treatment effectively reduced the recurrence rate compared to the acitretin group and resulted in a significant decrease in adverse reactions, indicating that the oxymatrine treatment effectively improved severe plaque psoriasis [130].

The bioactive constituents of traditional Chinese medicine have shown broad potential in restoring the skin barrier, achieving protection and repair through various mechanistic targets. Future research should further explore the specific molecular mechanisms and clinical application potential of these bioactive constituents to provide more options for treating skin barrier dysfunction. Table 6 summarizes the pharmacological effects and applications

## 6. Summary and Outlook

Human skin is often considered the primary defense mechanism and barrier against various infections affecting the body. Maintaining healthy skin is crucial for overall well-being, and it is achievable through a combination of modern allopathic and natural remedies. Common skin conditions like AD, psoriasis, eczema, acne, and chloasma pose significant healthcare challenges. Plant and animal extracts have proven effective in treating skin infections.

With the advancement of science and technology, the clinical demand for traditional Chinese medicine has significantly increased. As a vital component of traditional medicine, traditional Chinese medicine has amassed extensive experience in the treatment of skin diseases. Numerous traditional Chinese medicines exhibit anti-inflammatory, antibacterial, and anti-allergic properties, effectively alleviating the symptoms of skin conditions and enhancing overall skin health. Consequently, natural herbal remedies for skin diseases have emerged as crucial elements in the management of skin infections. Traditional Chinese medicine extracts have made substantial contributions to human healthcare. Certain extracts and compound preparations derived from traditional Chinese medicine, such as berberine, coix seed, and angelica dahurica, have demonstrated their efficacy in treating skin diseases through clinical trials. These traditional remedies are applicable in the treatment of common skin disorders, including eczema, psoriasis, acne, and urticaria. Additionally, traditional Chinese medicine possesses several unique advantages in addressing skin diseases. Primarily, it emphasizes holistic conditioning, which can enhance both the internal and the external environments of the human body, thereby boosting immunity and reducing the incidence and recurrence of skin diseases. Furthermore, traditional Chinese medicine is characterized by its diversity, allowing for personalized treatment approaches tailored to individuals’ constitutions and conditions, ultimately improving therapeutic outcomes.

Recent years have seen significant advancements in research into the functional elements in traditional Chinese medicine, with an increasing number of components showing potential in preventing and treating skin ailments. These factors, which are integral to traditional medicine, are gaining recognition in modern medical practice. Future studies should focus on understanding the mechanisms of action of these functional components and confirming their efficacy through clinical trials. This will promote the utilization and advancement of traditional Chinese medicine in treating skin diseases.

## Figures and Tables

**Figure 4:**
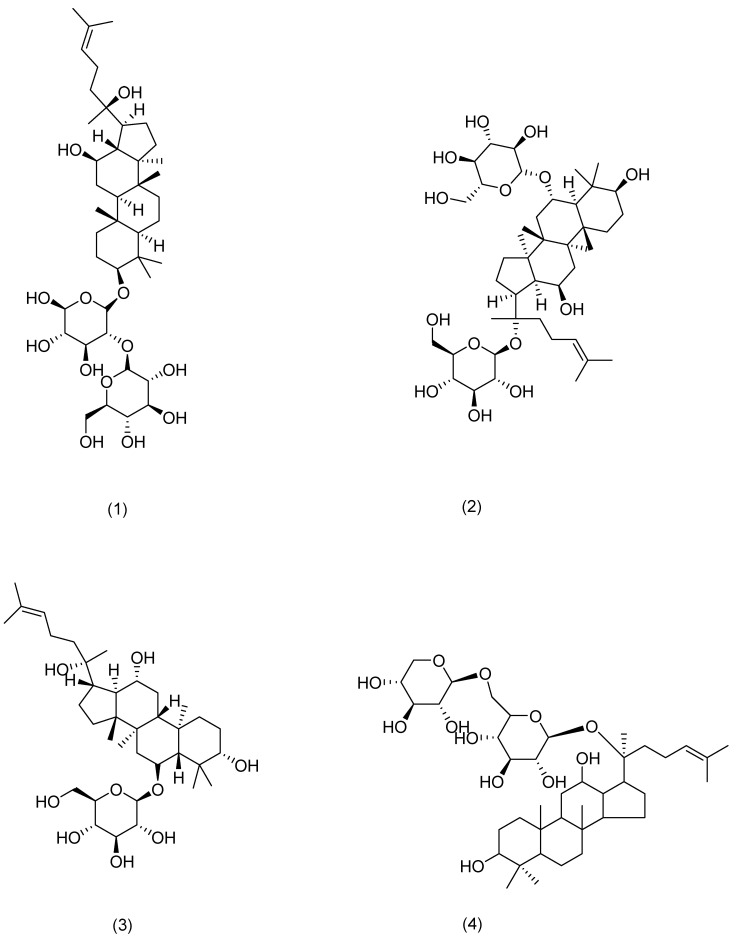
Structural formula of ginsenosides.

**Figure 5:**
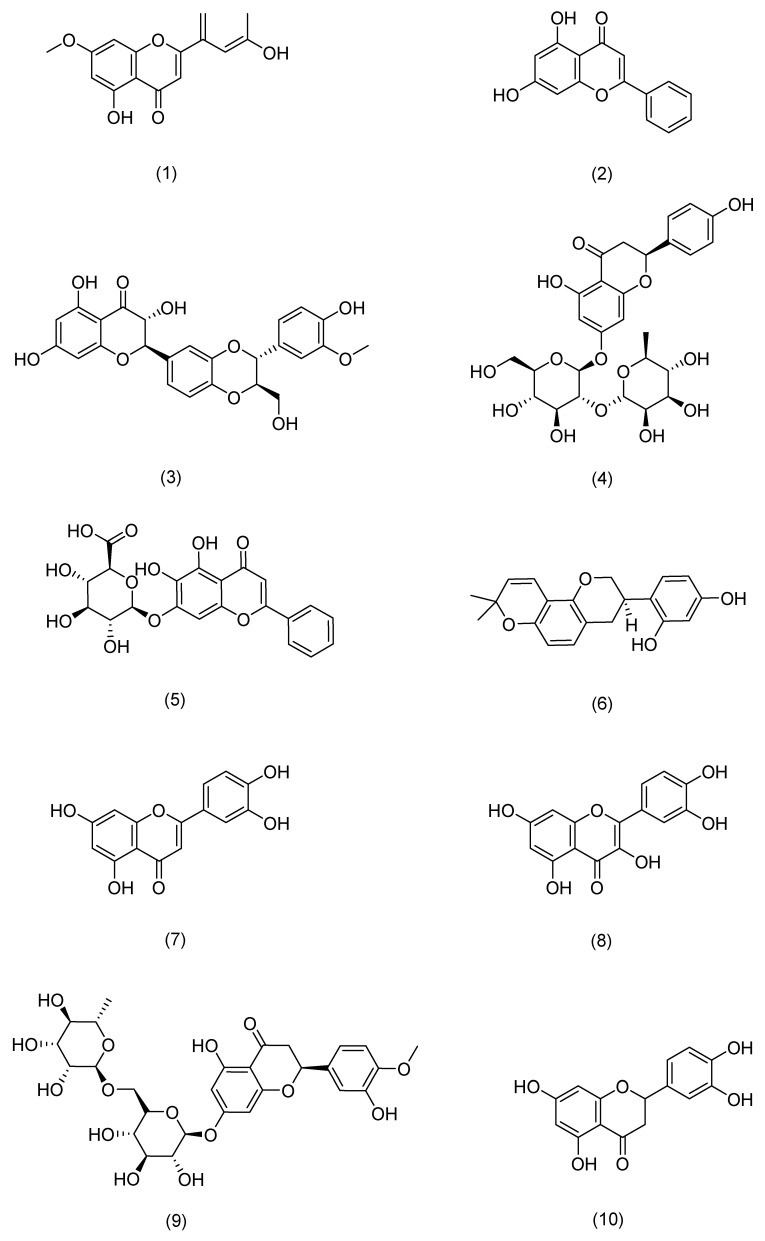

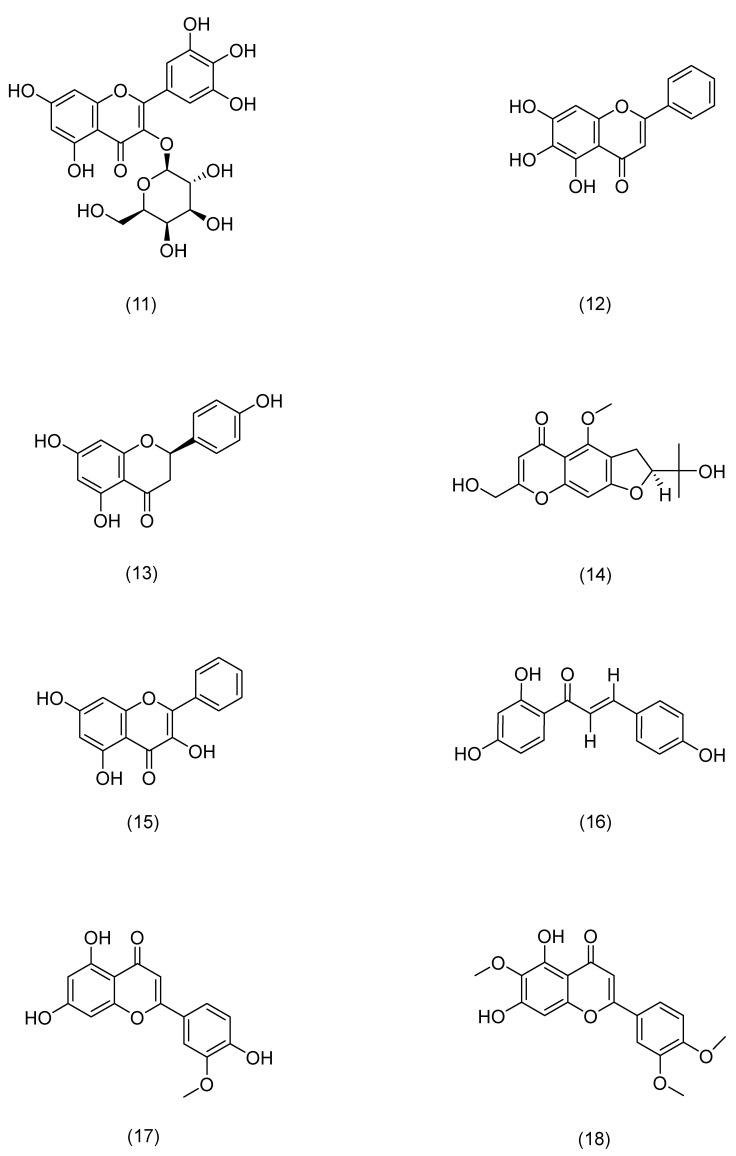
Structural formula of flavonoids.

**Figure 6:**
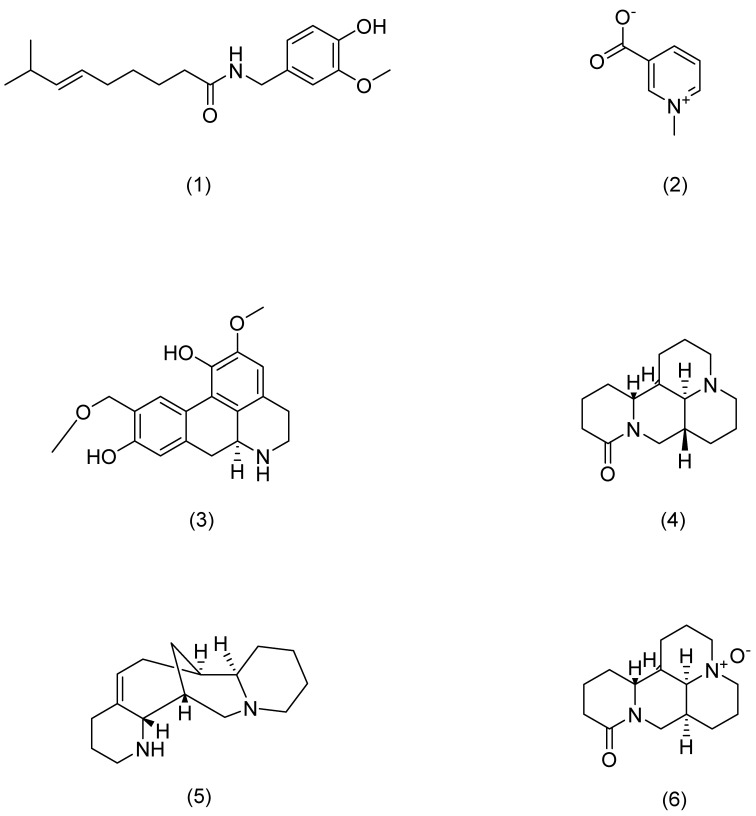
Structural formula of alkaloids.

**Figure 7:**
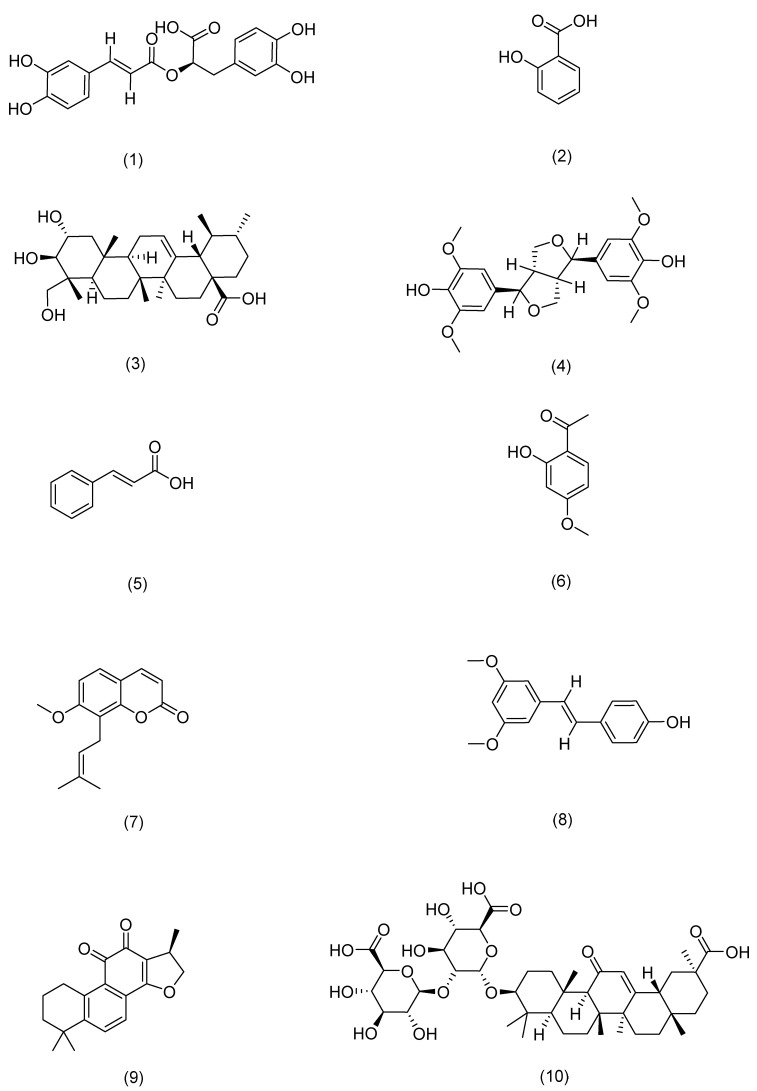

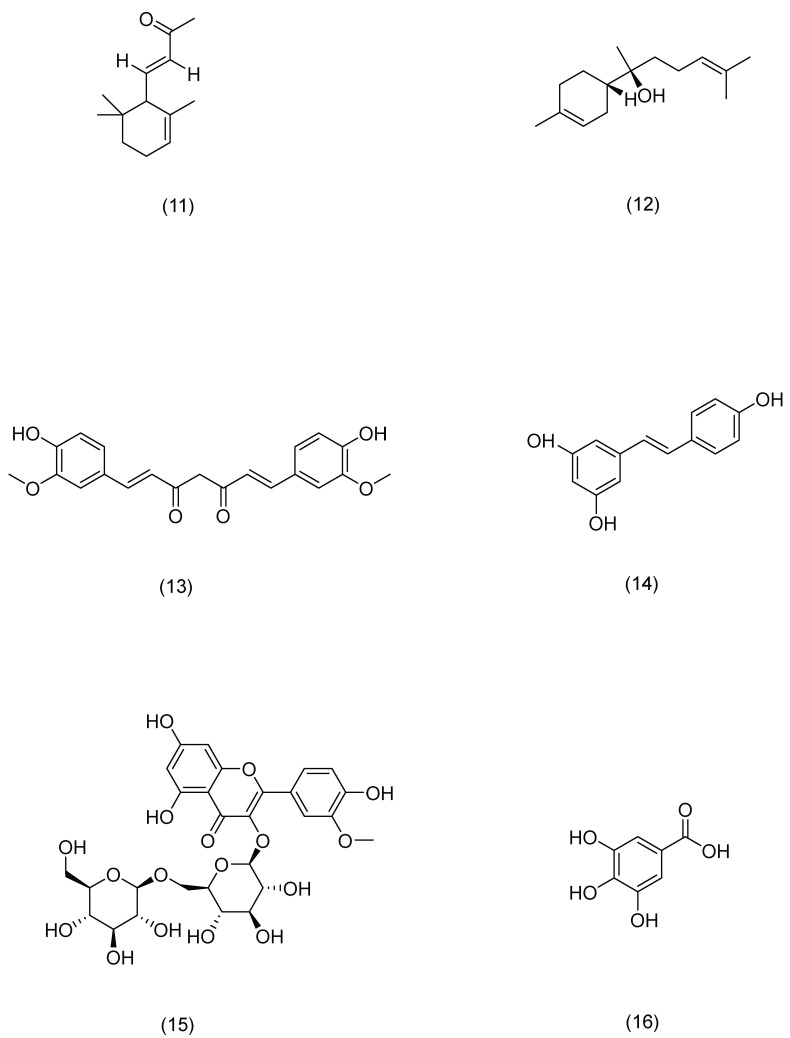
Structural formula of aother.

**Table 1:** Ginsenosides’ mechanisms of action.

Serial Number	Ginsenosides	Source	Latin Name	Structural Formula	Pathway	Pharmacological Effects	Mechanism	Literature
1	Ginsenoside Rg3	Ginseng	*Panax ginseng* C. A. Mey.	Compound (**1**) in Figure 4	MDM2/HIF1α	anti-inflammatory, antitumor, anti-photoaging [60,61]	Restoration of mitochondrial ATP and membrane potential inhibited the production and mRNA expression levels of TSLP and VEGF in activated HMC-1 cells. Rg3 downregulates MDM2 expression levels.	HanN R [53]
2	Ginsenoside Rg1			Compound (**2**) in Figure 4	NF-κB	anti-inflammatory [62]	Downregulation of NF-κB signaling pathway eliminates psoriasis-like dermatitis.	Shi Q and Park E K [62,63]
3	Ginsenoside Rh1			Compound (**3**) in Figure 4	NF-κB	anti-anaphylaxis, anti-inflammatory, antitumor [63]	Inhibition of the protein expression of iNOS and COX-7.	Park E K [63]
4	Ginsenoside C-Mx	Notoginseng	*Panax notoginseng* (Burkill) F. H. Chen ex C. H. Chow	Compound (**4**) in Figure 4	TGF-β, Smad, AP-1	anti-inflammatory, antioxidant, anti-photoaging [59]	Inhibition of intracellular ROS, MMP-1, and IL-6 expression, acceleration of the secretion of TGF-β and type I procollagen.	Liu X Y [59]

**Table 2:** Flavonoids’ mechanisms of action.

Serial Number	Flavonoids	Source	Latin Name	Structural Formula	Pathway	Pharmacological Effects	Mechanism	Literature
1	7-O-methylluteolin	Hawthorn berry	*Crataegus pinnatifida* Bunge	Compound (**1**) in Figure 5	Nrf2,HO-1	anti-inflammatory, antioxidant [72]	Reduces serum immunoglobulin E (IgE) and interleukin-4 (IL-4) levels.	Kim T Y [72]
2	Chrysin	Passiflora caerulea	*Passiflora caerulea* L.	Compound (**2**) in Figure 5	MAPK,JAK-STAT, NF-κB	anti-inflammatory, antioxidant [73]	Reduces TNF-α-, IL-17A-, and IL-22-induced release of CCL20 and antimicrobial peptides from epidermal keratinocytes.	Li H J [73]
3	silibinin	Milk thistle	*Silybum marianum* (L.) Gaertn.	Compound (**3**) in Figure 5	NF-κB	antioxidant [74]	Inhibits intracellular ATP and GSH consumption, ROS production, and membrane lipid peroxidation.	Svobodová A [74]
4	Naringin	Tangerine	*Citrus reticulata* Blanco	Compound (**4**) in Figure 5	MAPK,p38	antioxidant, anti-inflammatory [75]	Inhibits ROS production, COX-2 overexpression, and strong inflammatory response.	Ren X [75]
5	Baicalin	Skullcap	*Scutellaria baicalensis* Georgi	Compound (**5**) in Figure 5	TLR4	antioxidant, anti-inflammatory, anti-photoaging [76]	Protective effects on UVA-induced oxidative damage and inflammation in mouse skin by up-regulating IL-12 and IL-23 cytokines.	Sherwani M A [76]
6	glabridin	Licorice	*Glycyrrhiza uralensis* Fisch.	Compound (**6**) in Figure 5	MAPK, NF-κB	anti-inflammatory, anti-photoaging [77]	Inhibits the production of inflammatory cytokines such as TNF-α, IL-6, and IL-10.	Zhang C [77]
7	Luteolin	Callicarpa nudiflora	*Callicarpa nudiflora* Hook. & Arn.	Compound (**7**) in Figure 5	NF-κB,JAK-STAT,TLR	antioxidant, anti-inflammatory, anti-photoaging [78]	Inhibits pro-inflammatory mediators IL-1β, IL-6, IL-8, IL-17, IL-22, TNF-α, and COX-2.	Gendrisch F [78]
8	Quercetin	Honeysuckle	*Lonicera japonica* Thunb.	Compound (**8**) in Figure 5	PKCδ,JAK2	antioxidant, against cancer, anti-inflammatory, antidiabetic [79]	Inhibition of UV-induced MMP-1 and COX-2 expression.	Shin E J [79]
9	Hesperidin	Tangerine	*Citrus reticulata* Blanco	Compound (**9**) in Figure 5	MAPK	antioxidant, anti-inflammatory, immunomodulatory [80]	Reduces expression of MMP-9 and pro-inflammatory cytokines.	Lee H J [80]
10	eriodictyol	Lemon	*Citrus × limon* (L.) Osbeck	Compound (**10**) in Figure 5	MAPK	anti-inflammatory, anti-photoaging [81]	Enhances cell proliferation, reduces intracellular ROS production, downregulates the expression of inflammatory factors and MMP-1, and upregulates the expression of Timp1 and Col1.	Nisar M F [81]
11	Myricetin 3-O-β-d-galactopyranoside	Lemons	*Citrus × limon* (L.) Osbeck	Compound (**11**) in Figure 5	MAPK,AP-1,TGFβ/Smad	anti-inflammatory, anti-photoaging [82]	Downregulates the expression of MMP-1, but also reduces the protein levels of MMP-9 and MMP-3.	Oh J H [82]
12	Baicalein	Skullcap	*Scutellaria baicalensis* Georgi	Compound (**12**) in Figure 5	TRPV1-Ca-ERK	antioxidant, anti-photoaging [83]	Inhibits MMP-1 expression.	Huang K F [83]
13	Naringenin	Tangerine	*Citrus reticulata* Blanco	Compound (**13**) in Figure 5	ERK2	anti-photoaging [84]	Downregulates AP-1 transactivation and MMP-1 expression.	Jung S K [84]
14	Cimifugin	Cimicifuga	*Actaea cimicifuga* L.	Compound (**14**) in Figure 5	NF-κB (IκB, p65), MAPK (JNK, ERK, p38)	anti-inflammatory, antioxidant [69]	Inactivates the NF-κB/MAPK signaling pathway to prevent oxidative stress and inflammation in psoriasis-like pathogenesis.	Liu A [69]
15	galangin	Galangal	*Alpinia officinarum* Hance	Compound (**15**) in Figure 5	NF-κB,Nrf2	anti-inflammatory, against cancer [66]	Downregulates NF-κB and activates the Nrf2 signaling pathway to improve skin inflammation.	Sangaraju R [66]
16	Isoliquiritigenin	Licorice	*Glycyrrhiza uralensis* Fisch.	Compound (**16**) in Figure 5	CD177, JAK2, STAT	anti-inflammatory [85]	Downregulates the expression of IL-4, IL-6, IgE, and TSLP.	Wu Q [85]
17	Chrysoeriol	Cardiospermum halicacabum	*Cardiospermum halicacabum* L.	Compound (**17**) in Figure 5	NF-Κb, STAT3	antioxidant, anti-inflammatory [65]	Reduces protein levels of iNOS, COX-2, IL-6, IL-1β, and TNF-α.	Wu J Y [65]
18	Eupatilin	Mugwort leaves	*Artemisia argyi* H. Lév. & Vaniot	Compound (**18**) in Figure 5	P38MAPK, NF-κB	anti-inflammatory [68]	Inhibits the excessive proliferation of LPS-stimulated HaCaT cells and reduces the levels of TNF-α, IL-6, IL-23, and IL-17 in serum.	Bai D [68]

**Table 3:** Alkaloids’ mechanisms of action.

Serial Number	Alkaloids	Source	Latin Name	Structural Formula	Pathway	Pharmacological Effects	Mechanism	Literature
1	Capsaicin	Chili pepper	*Capsicum annuum* L.	Compound (**1**) in Figure 6	TRPV1	anti-inflammatory [88]	Blocks activation of IL-23/IL-17.	Chan T C [88]
2	Trigonelline	Fenugreek	*Trigonella foenum-graecum* L.	Compound (**2**) in Figure 6	PERK	anti-inflammatory, antioxidant, anti-photoaging [93]	Attenuates oxidative stress-mediated ER-stress and restores Ca2+ homeostasis.	Lone A N [93]
3	Norisoboldine	Lindera aggregata	*Lindera aggregata* (Sims) Kosterm.	Compound (**3**) in Figure 6	NFAT	anti-inflammatory [91]	Reduces mRNA levels of INF-γ, TNF-α, IL-4, and IL-6.	Gao S [91]
4	Aloperine	Sophora	*Sophora alopecuroides* L.	Compound (**4**) in Figure 6	STAT3	anti-inflammatory [92]	Inhibits Th17 differentiation and dendritic cell activation, and reduces the expression and secretion of pro-inflammatory cytokines.	Zhou H F [92]
5	Matrine	Sophora flavescens	*Sophora flavescens* Aiton	Compound (**5**) in Figure 6	Hsp6,NF-κB	anti-inflammatory [89]	Inhibits inflammatory cytokine secretion.	Huang P [89]
6	Oxymatrine	Sophora flavescens	*Sophora flavescens* Aiton	Compound (**6**) in Figure 6	IFN-γ	anti-inflammatory [87]	Activates p1, JNK, and Akt and downregulates MDC, ICAM-1, and SOCS38 to repair skin barrier.	Gao C J [87]

**Table 4:** Carbohydrates’ mechanisms of action.

Serial Number	Carbohydrates	Source	Latin Name	Pathway	Pharmacological Effects	Mechanism	Literature
1	Lycium barbarum polysaccharide	Lycium chinense	*Lycium chinense* Mill.	Nrf2/ARE, p38 MAPK	antioxidant, anti-inflammatory, anti-photoaging [97]	Scavenging ROS and reducing DNA damage, inhibiting caspase-3 activation and MMP-9 expression.	Li H [97]
2	Aloe polysaccharide	Aloe vera	*Aloe vera* (L.) Burm. f.	Keap1/Nrf2/ARE	antioxidant, anti-photoaging [99]	Improving cell antioxidant capacity to improve cell viability and proliferation to protect cells	Yuan L [99]

**Table 5:** Compounds’ mechanisms of action.

Serial Number	Compound	Source	Latin Name	Structural Formula	Pathway	Pharmacological Effects	Mechanism	Literature
1	Rosmarinic acid	Rosemary	*Rosmarinus officinalis* L.	Compound (**1**) in Figure 7	PLC-γ1,ITK	anti-inflammatory, antioxidant, antibacterial [109]	Activates CD4(+) T cells and significantly inhibits IFN-γ and IL-4 production.	Jang A H [109]
2	Salicylic acid	Red clover	*Trifolium pratense* L.	Compound (**2**) in Figure 7	SREBP-1,NF-κB	anti-inflammatory [110]	Reduces lipogenesis in sebocytes and suppresses inflammation in cells.	Lu J [110]
3	Asiatic acid	Centella	*Centella asiatica* (L.) Urb.	Compound (**3**) in Figure 7	NF-κB,MAPK	anti-inflammatory, immunomodulatory [111]	Downregulates the mRNA expression levels of AD-related cytokines.	Moon G H [111]
4	Syringaresinol	Tricyrtis pilosa	*Tricyrtis pilosa* Wall.	Compound (**4**) in Figure 7	MAPK,AP-1	anti-inflammatory, anti-photoaging [112]	Inhibits MMP-1 upregulation.	Oh J H [112]
5	Trans-cinnamic acid	Cinnamon	*Cinnamomum cassia* (L.) D. Don	Compound (**5**) in Figure 7	AP-1,Nrf2	antioxidant, anti-photoaging [113]	Inhibits MMP-1/-3 activation.	Hseu Y C [113]
6	Paeonol	Peony	*Paeonia × suffruticosa* Andrews	Compound (**6**) in Figure 7	DLD,Nrf2,ARE,MAPK,AP-1	anti-photoaging [114]	Inhibits the phosphorylation of mitogen-activated protein kinase and activator protein 1, resulting in the degradation of type I procollagen.	Sun Z [114]
7	Osthole	Cnidium monnieri	*Cnidium monnieri* (L.) Spreng.	Compound (**7**) in Figure 7	PI3K,Akt	anti-inflammatory, anti-viral, anti-anaphylaxis [115]	Controls the expression of tight-junction proteins in the skin, and can improve skin barrier damage.	Chen J R [115]
8	Pterostilbene	Pterocarpus indicus	*Pterocarpus indicus* Willd.	Compound (**8**) in Figure 7	Nrf2,ARE	antioxidant, anti-inflammatory, anti-cancer [116]	Induces the expression of antioxidant enzymes, thereby preventing UVB-induced oxidative stress.	Li H [116]
9	Cryptotanshinone	Salvia	*Salvia miltiorrhiza* Bunge	Compound (**9**) in Figure 7	AMPK,SIRT1,PGC-1α	anti-inflammatory, antioxidant, anti-tumor [117]	Inhibits ROS production and reduces DNA damage, reduces mitochondrial dysfunction, and promotes mitochondrial biogenesis.	Guo K [117]
10	Glycyrrhizinate	Licorice	*Glycyrrhiza uralensis* Fisch.	Compound (**10**) in Figure 7	MAPK,NF-κB	anti-photoaging, antioxidant [118]	Prevents epidermal hyperplasia, lymphocyte infiltration, and the expression of several inflammatory proteins: p38, JNK, COX-2, NF-κB, and ICAM-1.	Farrukh M R [118]
11	α-ionone	Raspberry	*Rubus idaeus* L.	Compound (**11**) in Figure 7	cAMP	anti-photoaging [119]	Improves cell proliferation and migration, as well as HA and HBD-2 production in HaCaT cells.	Yang D [119]
12	(-)-α-bisabolol	Chamomile	*Matricaria chamomilla* L.	Compound (**12**) in Figure 7	MAPK,NF-κB	anti-inflammatory [120]	Reduces levels of beta-hexosaminidase, histamine, and TNF-alpha.	Li G [120]
13	Curcumin	Turmeric	*Curcuma longa* L.	Compound (**13**) in Figure 7	TGF-β, Smad2/3,Smad7	anti-photoaging, antioxidant [121]	Restores the activity of antioxidant enzymes and attenuates ER stress, inflammation, and apoptosis signals.	Liu X [121]
14	Resveratrol	Knotweed	*Reynoutria japonica* Houtt.	Compound (**14**) in Figure 7	Nrf2	anti-photoaging, antioxidant [122]	Degrades Keap1 protein and promotes Nrf2 accumulation in the nucleus.	Liu Y [122]
15	Astragaloside	Astragalus	*Astragalus membranaceus* (Fisch.) Bunge	Compound (**15**) in Figure 7	TLR4,NF-κB	anti-photoaging, antioxidant [123]	Inhibits the production of pro-inflammatory cytokines and the expression of TLR4 and its downstream signaling molecules, NF-κB, iNOS, and COX-2 proteins.	Wang J [123]
16	Gallic acid	Cornus officinalis	*Cornus officinalis* Siebold & Zucc.	Compound (**16**) in Figure 7	Nrf2	anti-photoaging [101]	Decreases the mRNA and protein expression of keratin 16 and keratin 17, which are the markers of psoriasis.	Zhang J [101]

**Table 6:** Pharmacological effects and applications.

Serial Number	Compound	Source	Pharmacological Effects	Application
1	Luteolin	Licorice	Anti-tumor, anti-inflammatory, anti-viral, immunomodulatory, hepatoprotective, memory enhancement, and neuroprotective effects [131]	Psoriasis [132]
2	Hypericin	Hypericum perforatum	Anti-inflammatory, antidepressant, antibacterial [133]	Psoriasis [134]
3	Baicalin	Skullcap	Liver protection, anti-tumor, antibacterial, antiviral, antioxidant effects [135]	Psoriasis [136]
4	Indigo	Indigo	Antioxidant, anti-inflammatory [137]	Psoriasis [138]
5	Paeoniflorin	Wood Dan	Hepatoprotective, choleretic, anti-inflammatory, antioxidant, neuroprotective, anti-diabetic, anti-apoptotic, and anti-tumor [139]	Psoriasis [140]
6	Quercetin	Houttuynia cordata	Anti-inflammatory, antibacterial, antiviral, antioxidant, and antitumor [141]	Psoriasis [142], atopic dermatitis [143]
7	Galangin	Plantago	Anti-ulcer, anti-cancer, immune modulation, anti-infection, anti-inflammatory, and antioxidant [144]	Psoriasis [66]
8	Cimicithin	Cimicifuga	Anti-inflammatory [145]	Psoriasis [69]
9	Ginsenoside Rg1	Ginseng	Anti-inflammation, anti-fatigue, and immune regulation [146]	Psoriasis [62]
10	Kaempferol	Cassia	Antibacterial, anti-inflammatory, antioxidant, antimalarial, and antimutagenic activity [147]	Acne [148]
11	Naringenin	Fenugreek	Anti-cholesterol, anti-tumor, and anti-inflammatory [149]	Atopic dermatitis [150]
12	Rosmarinic acid	Rosemary	Antibacterial, anti-inflammatory, antioxidant, anti-apoptotic, anti-tumorigenic, anti-nociceptive, and neuroprotective properties [151]	Atopic dermatitis [109]
13	Epigallocatechin gallate	Green tea	Antioxidant, anticancer, hypoglycemic, antibacterial, antiviral, and neuroprotective [152]	Acne [153]
14	Capsaicin	Chili	Anti-inflammatory, analgesic, anticonvulsant,a and neuroprotective effects [154]	Psoriasis [88]
15	Salicylic acid	Red clover	Antioxidant, anticancer, and blood sugar regulation [155]	Acne [110]
16	Isoliquiritigenin	Licorice	Anti-inflammatory, anti-ulcer, antibacterial, and anti-cancer [156]	Atopic dermatitis [85]
17	Licorice acid	Licorice	Antioxidant, anticancer, and diuretic [157]	Psoriasis [70]
18	Asiatic acid	Centella	Sedative, analgesic, antidepressant, antibacterial, antiviral, and immunomodulatory effects [158]	Atopic dermatitis [111]
19	Chioku Shiaki	Citrus aurantium	Antioxidant [159]	Atopic dermatitis [160]
20	Isozoranthin	Mugwort leaves	Antibacterial, antiviral, hemostatic, antitumor, hepatoprotective, analgesic, anti-inflammatory, and antioxidant [161]	Psoriasis [68]

## Abbreviations

TCM	Traditional Chinese Medicine	IL-13	interleukin-13
TEWL	transepidermal water loss	TCS	topical corticosteroids
TJs	tight junctions	TCI	calcineurin inhibitors
TLR	toll-like receptor	pDCs	plasmacytoid dendritic cells
NMFs	natural moisturizing factors	IFN	interferon
SC	stratum corneum	mDCs	myeloid dendritic cells
UV	ultraviolet	IL-17	interleukin-17
KLK	kallikrein-related peptidase	IL-1	interleukin-1
SG	stratum granulosum	IL-6	interleukin-6
AMPs	antimicrobial peptides	CXCL1	chemokine (C-X-C motif) ligand 1
hBDs	human beta-defensins	CCL20	chemokine ligand 20
LCs	langerhans cells	PASI	Psoriasis Area and Severity Index
DCs	dermal dendritic cells	TH2	T helper cell 2
IL-7	interleukin-7	TH17	T helper cell 17
IL-15	interleukin-15	TH9	T helper cell 9
TGF-β	transforming growth factor b	TNF-α	tumor necrosis factor-α
IL-34	interleukin-34	TNCB	2,4,6-trinitrochlorobenzene
PAMPs	pathogen-associated molecular patterns	DNCB	1-Chloro-2,4-dinitrobenzene
DAMPs	damage-associated molecular patterns	CK2α	casein kinase 2
NF-κB	nuclear factor kappa-B	GSH	glutathione, reduced
AD	atopic dermatitis	BALB/c	laboratory-bred strain
TSLP	thymic stromal lymphopoietin	IVL	involucrin
IgE	immunoglobulin E	AQP3	aquaporin 3
IL-4	interleukin-4	ATP	adenosine triphosphate
ELISA	enzyme-linked immunosorbent assay	COX-7	cyclooxygenase-7
HaCaT	human keratinocyte cells	COX-2	cyclooxygenase-2
UVB	ultraviolet B	GAL	galangin
SOD	superoxide dismutase	GST	glutathione S-transferase
FLG	filaggrin	GR	glutathione reductase
Cldn-1	claudin-1	LPS	lipopolysaccharides
NHDF	human dermal fibroblasts	GL	glycyrrhizic acid
AP-1	activator protein-1	MCP-1	monocyte chemotactic protein-1
HO-1	heme oxygenase-1-IN-1	TLR	toll-like receptors
NQO-1	NAD (P)H quinone dehydrogenase 1	SOCS1	suppressor of cytokine signaling 1
iNOS	inducible nitric oxide sythase	PCR	polymerase chain reaction
ROS	reactive oxygen species	SPINK5	serine protease inhibitor Kazal type-5
IL-1β	interleukin-1β	KLK7	kallikrein-related peptidase 7
IMQ	imiquimod	LBP	lycium barbarum polysaccharide
CAT	catalase	APS	astragalus polysaccharide
GSH	glutathione, reduced	AP	aloe polysaccharide
Vit-C	vitamin C	FCM	flow cytometry
ICAM-1	intercellular cell adhesion molecule-1	Bcl-2	B-cell lymphoma-2
IFN-γ	interferon-γ	GSTP1	glutathione S-transferase Pi
TACR1	tachykinin receptor 1	UA	ursolic acid
UVA	ultraviolet A	SREBP-1	sterol regulatory element-binding protein-1
PKCδ	protein kinase C-δ	RANTES	recombinant human C-C motif chemokine 5
CD177	CD177 Molecule	IP-10	recombinant human C-X-C motif chemokine 10
MDC	monodansylcadaverine	MIP-3a	macrophage Inflammatory Protein-3
NFAT	nuclear factor of activated T cells	PI3K	phosphatidylin-ositol-3-kinase
PMA	phorbol 12-myristate 13-acetate	SIRT1	silent information regulator family protein 1
GSO	ginseng oligosaccharide	HBD-2	human β-defensin-2
KLK5	kallikrein-related peptidase 5	SCORAD	SCORing Atopic Dermatitis
DSG1	desmoglein 1	INM-A	Indigo Naturalis
NFG	nerve growth factor	PCNA	proliferating cell nuclear antigen
MTT	thiazolyl blue	TUNEL	terminal deoxynucleotidyl transferase-mediated dUTP nick labeling
PI	propidium iodide	PGC-1α	peroxisome proliferators-activated receptor γ coactivator lalpha
Bax	BCL2-associated X	OXPHOS	oxidative phosphorylation
GCLC	glutamate cysteine ligase catalysis	Ki-67	large (395 kDa) nuclear protein
NFG	nerve growth factor	ITK	interleukin-2-inducible T-cell kinase
ONA	oleanolic acid	CD4(+)	cluster of differentiation 4 receptors
TARC	thymic and activating regulatory chemokine	MCP-1	monocyte chemotactic protein-1
